# Comparative Outcomes of Levetiracetam and Phenobarbital Usage in the Treatment of Neonatal Seizures: A Retrospective Analysis

**DOI:** 10.3390/healthcare12070800

**Published:** 2024-04-07

**Authors:** Handan Hakyemez Toptan, Nazmiye Nilgun Karadag, Sevilay Topcuoglu, Elif Ozalkaya, Emre Dincer, Hakan Cakir, Asli Okbay Gunes, Guner Karatekin

**Affiliations:** Department of Neonatology, University of Health Sciences, Zeynep Kamil Maternity and Children’s Disease Health Training and Research Center-Istanbul, 34668 Istanbul, Turkey; fbnilg2010@gmail.com (N.N.K.); sevilaymd@yahoo.com (S.T.); elifozalkay@gmail.com (E.O.); asliokbay@gmail.com (A.O.G.);

**Keywords:** neonatal seizures, phenobarbital, levetiracetam, adverse events, safety profile, mortality rate, discharge rate

## Abstract

Objectives and Aim: The primary aim of this study was to conduct a comparative analysis of the safety and efficacy of levetiracetam (LEV) and phenobarbital (PB) as first-line treatments for neonatal seizure management. This study was designed to measure and compare the incidence of adverse effects and to determine the discharge and mortality rates associated with the use of these antiseizure medications (ASMs). Through this comparison, this research sought to provide insights to optimise care for neonates experiencing seizures. Materials and Methods: This retrospective cohort study evaluated 104 neonates treated for seizures at Zeynep Kamil Hospital from 2015 to 2020 after excluding those on non-PB/LEV antiseizure medications. Seizures were characterised using electroencephalogram (EEG) and categorised according to aetiology and frequency. Treatment efficacy was gauged by seizure cessation, as confirmed using EEG. Adverse effects and demographic data were recorded. Statistical analyses were conducted using SPSS, employing the Shapiro–Wilk, independent *t*-test, Mann–Whitney U test, and chi-square test, with a significance threshold of *p* < 0.05. Results: Overall, 104 neonates treated with first-line ASM were evaluated for efficacy; PB was administered in 68.26% of the cases, while LEV was utilised in 31.74%. The total complete response rate was 40.38%, with no significant difference between the PB and LEV groups (*p* = 0.309). The incidence rate ratios (IRRs) demonstrated that seizure frequency profoundly influenced treatment effectiveness, with IRRs of 2.09 for rare seizures, 3.25 for frequent seizures, and 4.01 for status epilepticus, indicating a higher treatment response rate with increasing seizure frequency. For second-line treatment, among a subset of 62 patients, PB had a slight, non-significant advantage over LEV, with an odds ratio of 1.09, suggesting a marginally better response to LEV. Adverse events were significantly more frequent in the PB group, affecting 19 of 67 neonates (28.36%), compared to only 2 of 71 neonates (2.82%) in the LEV group (*p* < 0.001). No significant difference was observed in the discharge rates between the two groups (PB, 67.61%; LEV, 75.76%; *p* = 0.674). Interestingly, the mortality rate was significantly higher in the LEV group (45.45%) than that in the PB group (22.54%; *p* = 0.045). Conclusion: This study underscores LEV’s superior safety profile over PB in neonatal seizure management, evidenced by a significantly lower rate of adverse events. PB seems to be more effective in the second-line treatment of neonatal seizures. Despite the lack of significant differences in the discharge rates, the higher mortality rate associated with LEV warrants further investigation. These findings advocate the cautious selection of antiepileptic drugs in neonatal care, with a preference for LEV based on its safety profile.

## 1. Introduction

Neonatal seizures, a critical neurologic condition affecting 1–4 out of 1000 live births and significantly more prevalent in preterm infants, pose a substantial risk of adverse neurodevelopmental outcomes [1]. The treatment for these seizures remains a critical area of debate. Phenobarbital (PB), despite being the oldest and most commonly used first-line antiseizure medication, is only partially effective and can lead to detrimental effects such as neuronal apoptosis and synaptic development issues in neonates [2]. Consequently, there is a growing inclination towards third-generation antiseizure medications such as levetiracetam (LEV), which are associated with fewer adverse effects and a potentially better safety profile [3]. However, the lack of standardised treatment protocols underscores the urgent need for evidence-based guidelines for neonatal seizure management.

In the landscape of neonatal seizure management, there has been a notable shift over the past decade from the traditional use of PB to LEV, driven by LEV’s favourable pharmacokinetic profile, including linear clearance and minimal drug interactions, as opposed to PB’s complex clearance dynamics and numerous drug interactions [4]. Despite the increasing preference for LEV owing to its perceived safety and efficacy, robust evidence confirming its superiority over PB is still lacking. Recent studies have reported conflicting results, with some showing comparable effectiveness and others highlighting PB’s superior seizure control, but with a higher adverse effect profile [5,6]. The current literature underscores the necessity for a more in-depth comparative analysis to optimise treatment protocols for neonatal seizures, considering both the efficacy and safety of these medications.

Although neonatal seizures signal an underlying neurological disorder and can substantially impact long-term outcomes, current treatments offer varied results in terms of safety and efficacy. Advances in neonatal care and diagnostics have led to increased detection of these seizures, emphasising the need for treatment modalities that are not only effective but also safeguard the developing brain. Third-generation antiseizure medications, such as levetiracetam, have been welcomed as potential alternatives to phenobarbital because of their reduced risk of neurotoxic effects, which is a consideration of paramount importance given the susceptibility of the neonatal brain to injury. However, translation of these considerations into clinical practice requires rigorous evaluation through randomised controlled trials and observational studies to develop an evidence-based consensus for first-line therapies. A judicious approach that considers the unique pharmacokinetic and pharmacodynamic properties of antiseizure medications in neonates is essential, as underscored by recent meta-analyses which suggest that while newer medications are promising, they are not without their own risks and limitations [7,8].

The primary objective of this study was to conduct a thorough comparative evaluation of the safety profiles and therapeutic efficacy of levetiracetam (LEV) versus phenobarbital (PB) when used as first-line antiseizure medication in neonatal seizure management. This involved the analysis of retrospective patient data to identify potential differences in clinical outcomes, including the frequency and severity of adverse events, rate of seizure control, and total treatment success. We sought to contribute valuable evidence-based insights that could inform and refine clinical practice guidelines for the treatment of neonatal seizures.

## 2. Materials and Methods

### 2.1. Data Collection and Study Population

Prior to commencement of the study, the local ethics committee of the Zeynep Kamil Maternity and Children’s Disease Health Training and Research Hospital approved the study protocol (Approval Date: 17 March 2021, Reference No. 73). The retrospective nature of the study negated the need for informed consent from the parents of the neonates. This study adhered to the ethical standards of the 2013 Revised Declaration of Helsinki.

We retrospectively analysed 141 neonates treated at our hospital between 2015 and 2020. The inclusion criterion was a consensus on the clinical team. After excluding 37 patients who received non-PB/LEV antiseizure medications or had no confirmed electrographic seizures, 104 neonates were included in the final cohort. The cohort was divided into two groups based on the administration of antiseizure medication (groups PB and LEV) for the resolution of neonatal seizures.

Inclusion criteria were neonates at risk for seizures or those with suspected seizures (these terms refer to neonates who presented with clinical signs suggestive of seizures, such as abnormal movements or autonomic signs, which prompted further evaluation with EEG). Additional inclusion criteria were neonates who were not previously treated with antiseizure medications other than PB or LEV, with normal serum creatinine levels (≤1.6 mg/dL), and without seizures attributable to rectifiable metabolic derangements, such as hypoglycaemia or hypocalcaemia. Neonates with unconfirmed electrographic seizures were also excluded.

### 2.2. Characterisation of Neonatal Seizures

Neonatal seizures were characterised by the abrupt emergence of rhythmic EEG patterns persisting for at least 10 s, distinguished by alterations in amplitude, frequency, or spatial distribution. These parameters were measured using amplitude-integrated and standard EEG methods.

Electrographic seizures in neonates were defined as sudden, abnormal EEG events with a repetitive and evolving pattern with a peak-to-peak voltage of >2 mV and a duration of >10 s, while “evolving” refers to an unequivocal evolution in frequency, voltage, morphology, or location.

Seizure aetiology was classified based on the current framework for neonatal seizures and epilepsy syndromes such as hypoxic-ischaemic, structural vascular (including acute ischaemic stroke, haemorrhage, and other vascular-induced ischaemia), structural due to brain malformation, or genetic, infectious, metabolic, and unknown. We further divided seizure aetiology into acute symptomatic seizures, including hypoxic-ischaemic, structural vascular, infectious, and metabolic, and neonatal epilepsies, including structural seizures due to brain malformations and genetics.

Seizure frequency within the first 24 h was determined using clinical reports and the findings of continuous monitoring using EEG/amplitude-integrated EEG if available, and defined as rare (less than five seizures), frequent (>6 seizures), or status epilepticus, when the summed duration of seizures comprised 50% of a one-hour period.

### 2.3. Treatment Administration and Efficacy Assessment

Neonates exhibiting seizure activity were administered either LEV at a dosage of 30 mg/kg or PB at a dosage ranging from 15 to 20 mg/kg via a 15 min infusion. A subsequent observation period of 15 min was allocated to allow ASM to exert its therapeutic effects. In cases where seizures persisted or recurred 30 min after the initial infusion, an additional dose of LEV (30 mg/kg) was administered (capping at 60 mg/kg) or PB at incremental doses of 5 mg/kg every 15 min (not exceeding 40 mg/kg) (first-line treatment). Transition to alternative ASM occurred if seizures continued after the maximum dosage of the initial medication (second-line treatment).

Upon administration of the LEV loading dose, a maintenance regimen of 15–20 mg/kg was administered intravenously, twice daily. Conversely, following PB loading doses, maintenance therapy involved 3–5 mg/kg administered intravenously, once daily. The efficacy of the treatment was gauged by a complete cessation of seizures, as confirmed using EEG, negating the need for subsequent ASM administration. If two ASMs were used prior to seizure control, the latter was considered effective.

### 2.4. Adverse Effects and Demographic Correlation

Adverse effects identified and documented by the clinical team included any instances of hypotension, alterations in heart rate or respiratory function requiring supplemental oxygen or mechanical ventilation, irritability, sedation, or deviations in laboratory values attributable to ASM administration.

Demographic data (demographic data analysis included a breakdown by gender, exploring the prevalence of vascular structure issues, the incidence of genetic malformations, the rate of infection-driven seizures, and the total response to treatment) were recorded and compared between treatment groups.

### 2.5. Statistical Analysis

The data obtained in this study were statistically analysed using SPSS (version 25.0; SPSS for Windows^®^, Statistical Package for Social Sciences, IBM Inc., Chicago, IL, USA). The normality of distribution for continuous variables was tested using the Shapiro–Wilk test. For variables that followed a normal distribution, comparisons between the two groups were performed using an independent *t*-test. Non-normally distributed continuous variables were compared using the Mann–Whitney U test, which assesses differences between two independent groups when the dependent variable is either ordinal or continuous but not normally distributed. For categorical data, Fisher’s exact test was used instead of the chi-square test when the sample sizes were small or when the expected frequencies in any of the cells of a contingency table were below five. This test is a precise method for examining the associations between categorical variables. Descriptive statistics for continuous variables are presented as mean ± standard deviation (SD) and median with minimum–maximum (min–max) range, while categorical variables are shown as numbers and percentages. The statistical significance was set at *p* < 0.05.

## 3. Results

### 3.1. Demographic and Clinical Characteristics Compression

The analysis included 104 neonates treated for EEG-confirmed seizures. The sex distribution was balanced, with 56.73% of the patients being male and 43.27% being female. The mean birth weight was 1712.65 g, and the average gestational age was 31.05 weeks. The median maternal age was 31.5 years. The delivery modes included spontaneous vaginal delivery (37.50%) and caesarean section (61.54%).

Hypoxic-ischaemic factors were identified in 32.69% of patients, and vascular structural issues accounted for 25.00% of the seizures. Genetic malformations were less common in 10.58% of the cases. Infection-driven seizures comprised 10.58%, metabolic causes were observed in 0.96%, and the aetiology remained unknown in 18.27%.

Regarding treatment efficacy, PB was the first-line ASM in 68.26% of cases and LEV was used in 31.74% of cases. The complete response rate was 42 (40.38%), with no significant difference in the completion rate between the PB and LEV groups (*p* = 0.309). Our cohort had a discharge rate of 70.19% and a mortality rate of 29.81%. There were no significant differences in clinical characteristics between the neonate groups initially treated with either LEV or PB as an antiseizure medication. The detailed demographic and clinical characteristics of the study cohort are presented in Table 1.

### 3.2. Efficacy of PB and LEV in Initial and Subsequent Seizure Management

In this study, PB was the first-line ASM in 68.26% (71 of 104) of neonates, with 47.47% (47 of 71 patients) who did not achieve full seizure control.

Subsequent treatment showed that 68.08% of these patients received LEV again as the second-line treatment (32 of 47 patients), yielding a complete response rate of 78.12% (25 of 32 patients).

In contrast, 31.91% (15 of 32) switched to PB as the second-line treatment, with 80% (12) responding fully. Ten patients had incomplete seizure control after second-line LEV treatment.

In this study, PB was the first-line ASM in 31.74% (33 out of 104) of the cohort, and 21.13% (15 patients out of 33) had an incomplete initial response. Subsequent treatment showed that 33.33% of these patients (5 of 15) received PB again as the second-line treatment, yielding a 100% (5 of 5 patients) complete response rate. In contrast, 76.66% (10 of 15 patients) switched to LEV as the second-line treatment, with 80% (8 of 10 patients) responding fully. After the second-line treatments following PB were used as the first-line treatments, two patients remained with incomplete seizure control.

Following the first-line treatment, 25 (10 patients from PB to LEV, and 15 patients from LEV to PEB) changed treatments; a total of 12 patients exhibited persistent seizure symptoms after the second-line treatment.

**Figure 1:**
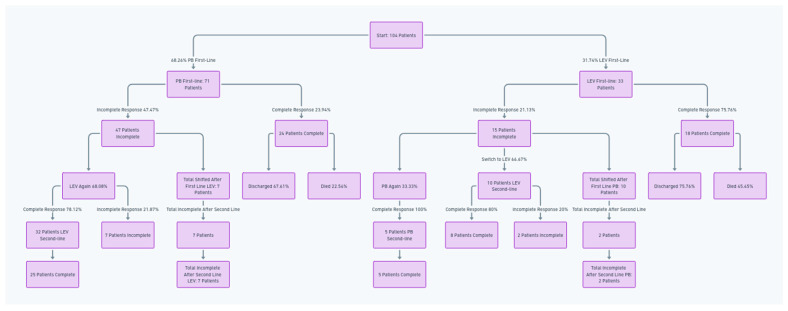
Flowchart of treatment progression and outcomes for neonates transitioning between first-line and second-line antiseizure medications.

A treatment flowchart is shown in Figure 1.

### 3.3. Determinates of Antiseizure Medication Efficacy Analysis

In the first-line treatment cohort, term neonates comprised the majority (79 patients), whereas preterm neonates comprised 25 patients. The aetiology of seizures varies, with hypoxic-ischaemic conditions being the most common. The incidence rate ratios (IRRs) indicated that seizure frequency was a significant factor in the effectiveness of treatment. Rare seizures showed an IRR of 2.09, frequent seizures had an IRR of 3.25, and status epilepticus had the highest IRR of 4.01 in the first line ASM. These numbers suggest that the more frequent the seizures, the greater the likelihood is of a response to treatment. In second-line treatment, a smaller sample of 62 patients was evaluated, with PB showing an odds ratio of approximately 1.09, indicating a slight improvement in response to LEV as the second-line therapy. Detailed multivariate analyses of neonatal seizure treatment are presented in Table 2.

### 3.4. Adverse Events

No instances of cardiopulmonary complications were reported, and no discontinuation of either PB or LEV owing to severe adverse effects occurred.

A statistically significant difference in adverse events was found between the treatments (*p* < 0.001), and adverse events were observed in 19 neonates treated with PB (28.36% of the PB group) compared to two neonates treated with LEV (2.82% of the LEV group). Adverse events observed in the PB group predominantly included respiratory depression, sedation, and hypotension, whereas in the LEV group, only a few cases involved mild sedation and irritability.

No significant differences were found according to treatment outcomes for discharge rate: 73 of 104 neonates were discharged, which reflects a discharge rate of 67.61% for the PB group (48 of 71 patients) and 75.76% for the LEV group (25 of 33 patients) (*p* = 0.674).

A significant difference was found according to treatment outcomes for mortality rate, with mortality rates of 22.54% in the PB group (16 of 71 patients) and 45.45% in the LEV group (15 of 33 patients) (*p* = 0.045).

## 4. Discussion

The overall treatment response to first-line antiseizure medications (ASMs) in neonates with seizures was relatively low (40.38%) and showed no substantial difference between levetiracetam (LEV) and phenobarbital (PB). The efficacy did not vary significantly with gestational age or aetiology. Notably, LEV demonstrated a more favourable safety profile, with adverse effects reported in only 2.82% of cases compared with those treated with PB. However, PB was potentially more effective as a second-line treatment, achieving a seizure cessation rate of up to 100% in some instances. This study suggests that while LEV could be considered a safe and viable first-line treatment option, PB may retain its efficacy, particularly in treatment-resistant neonatal seizures.

The comparative analysis of clinical features between neonates treated with PB and LEV revealed no significant sex distribution disparity, suggesting that sex does not influence treatment response. Birth weight and gestational week data did not show significant differences in treatment outcomes, indicating that these factors may not be pivotal in determining ASM efficacy.

The demographic and clinical characteristics observed during neonatal seizure management, such as sex distribution and gestational factors, have been examined in several studies. Research indicates that there is no significant correlation between the sex of neonates and the efficacy of treatment with ASMs. This aligns with findings from a Swedish study that assessed the relationship between maternal characteristics and birth weight for gestational age, which did not single out infant sex as a determinant of birth outcome [9]. Furthermore, birth weight and gestational age at birth, which are related to neonatal treatment outcomes, have been extensively studied. Variations in these factors do not necessarily predict the efficacy of ASMs, which resonates with the data suggesting that these individual characteristics may not significantly influence the effectiveness of neonatal seizure treatment. This is supported by studies that have not found substantial differences in neonatal metabolomic profiles based on the mode of delivery or gestational age, reinforcing the notion that these factors may not be critical determinants of the immediate metabolic status of newborns [2,3].

The current literature supports the finding that sex distribution, birth weight, and gestational week measurements may not significantly impact immediate treatment outcomes in neonatal seizure management. This information can be crucial for clinicians to understand that while these factors are important for neonatal health, they might not be decisive for the effectiveness of first-line ASM therapies.

In the context of our study, the efficacy of first-line anti-seizure medication (ASM) with phenobarbital (PB) showed a completion rate of 23.94%, whereas levetiracetam (LEV) achieved a notably higher completion rate of 75.76%. This finding aligns with observations from real-world data [10] yet diverges from reports of higher PB efficacy in other clinical trials [5]. This variance underscores the necessity of contextualising clinical data within the broader spectrum of existing research.

The seizure aetiology profile in our cohort, predominantly hypoxic-ischaemic, reflects a broad etiological spectrum that does not significantly affect the ASM responsiveness. This suggests that a potentially uniform therapeutic approach can be applied across different seizure aetiologies. LEV’s lower incidence of adverse events further supports its favourable profile as a potential primary treatment option.

Consistent with previous studies, our results showed no significant difference in response to first-line ASM between LEV and PB across various seizure aetiologies, including acute symptomatic seizures. The homogeneity in ASM response, despite etiological diversity, may validate the applicability of our findings to a wider clinical setting, particularly in the context of neonatal hypoxic-ischaemic encephalopathy [11,12]. Seizure frequency has emerged as a pivotal determinant of ASM efficacy. Our data revealed that neonates with infrequent seizures responded more favourably, suggesting that seizure frequency should be considered when devising treatment strategies. Higher seizure frequency was correlated with a reduced likelihood of response to first-line ASM, consistent with the established literature that correlates increased seizure frequency with diminished treatment success [2,13].

In summary, while PB remains a staple in neonatal seizure management, LEV has emerged as a potent, safe, and effective alternative first-line therapy. The comparable efficacy rates of LEV and PB underscore the viability of both medications in treatment protocols.

The absence of cardiopulmonary complications and the continuation of treatment without severe adverse effects for both PB and LEV were positive outcomes, indicating baseline safety in their use. However, the statistically significant difference in the occurrence of adverse events between the PB and LEV groups (28.36% vs. 2.82%, *p* < 0.001) was a critical finding. This disparity highlights LEV’s superior safety profile compared with PB, consistent with previous research that has similarly underscored LEV’s favourable adverse effect profile in neonatal populations [13,14].

Moreover, adverse events such as hypotension, respiratory suppression and sedation are particularly concerning in the neonatal context given the vulnerability of this population to such side effects. The significant prevalence of these adverse events in the PB group underscores the need for cautious PB use, especially considering the potentially grave implications of hypotension and respiratory depression in neonates with haemodynamic instability. The literature has documented the detrimental effects of PB-induced hypotension in neonates with hypoxic-ischaemic encephalopathy and post-cardiac surgery seizures, necessitating re-evaluation of its use in similar clinical scenarios [12,15].

In contrast, LEV’s safety profile is remarkably positive, with negligible serious adverse effects reported across multiple studies, even at high doses for seizure control [6,16]. This reinforces the potential of LEV as a safer alternative to PB in neonatal seizure management, a sentiment echoed by our findings and supported by recent randomised controlled trials (RCTs) [5,14].

Interestingly, no significant differences were found in the discharge rates between the two groups, suggesting that the decision regarding discharge readiness may not be directly influenced by the choice of AEDs. However, the significant difference in mortality rates (22.54% in the PB group vs. 45.45% in the LEV group, *p* = 0.045) is alarming and warrants further investigation. Several factors may contribute to this finding, such as the underlying severity of the treated conditions or other concurrent medical issues. Dosing could potentially influence outcomes; however, the study design and data limited the ability to draw definitive conclusions regarding causality. Further research is needed to explore these variables in greater detail. This discrepancy could suggest underlying differences in the severity of the conditions being treated, or potentially indicate other risk factors at play that are not directly related to AED choice.

The implications of this study are profound and advocate for a more judicious selection of AEDs for neonatal care. Although PB has been a longstanding option for neonatal seizures, its associated adverse effects, particularly in the context of haemodynamic instability, call for the re-evaluation of its use. Conversely, LEV has emerged as a safer alternative, with a significantly better adverse effect profile. Nevertheless, the higher mortality rate observed in the LEV group raises questions that exceed the scope of this study, highlighting the need for comprehensive research to fully understand these outcomes.

Our study corroborates the growing body of evidence favouring LEV over PB for neonatal seizure management, owing to its superior safety profile. These findings underscore the necessity for individualised treatment plans that consider the specific clinical context of each neonate to ensure an optimal balance between efficacy and safety.

### Study Limitations

Our investigation into the efficacy of PB and LEV in managing neonatal seizures, while comprehensive, has several limitations. The retrospective design and cohort size may have limited the identification of specific subpopulations that could benefit from particular ASM protocols. The exclusion of neonates with unverified electrographic seizures could introduce bias towards more pronounced seizure manifestations. Furthermore, the lack of randomisation and potential prescription bias in more severe cases could have influenced the perceived effectiveness of PB. The absence of real-time video-EEG monitoring also precludes the exact timing of seizure onset to treatment response, which is a critical factor in the effectiveness of ASM. Additionally, our cohort may not fully represent the wider neonatal population because of the exclusion of neonates treated with alternative ASMs and those without parental consent. Despite these limitations, our study provides valuable insights into real-world clinical settings, underscoring the need for further prospective randomised studies to refine neonatal seizure management strategies.

When discussing the comparative efficacy of PB and LEV as first-line antiseizure medications (ASMs) in neonatal seizure management, it is important to consider the broader context provided by contemporary research. The study at hand indicated that LEV was administered as the first-line ASM to a significant majority of the neonates; while many did not achieve complete seizure control, a substantial proportion showed a complete response upon subsequent treatments, either with the same ASM or after switching to PB. This nuanced response reflects the complex nature of neonatal seizures and the challenges in establishing universally effective treatment protocols. A closer look at the numbers reveals that a higher completion rate was achieved with LEV when used again as second-line treatment compared to those who switched to PB. This suggests that LEV not only serves as a viable first-line treatment, but also remains effective in subsequent interventions, which aligns with the existing literature that recognises LEV as a safe and generally well-tolerated option for neonatal seizures. For example, Sharpe et al. (2020) in a randomised controlled trial observed that LEV did not differ significantly from PB in terms of efficacy but was associated with fewer adverse effects [5].

In contrast, PB, which is less commonly used as the first ASM, displayed remarkable efficacy when repeated as second-line treatment, achieving a 100% completion rate in a small subset that received it again. This finding resonates with the historical view that PB is a cornerstone of seizure management, supported by the study’s findings that suggest its continued effectiveness, particularly in resistant cases.

The present study also touches upon the impact of seizure frequency on treatment outcomes, where higher seizure frequency correlated with reduced response rates to first-line ASM. This is consistent with previous studies indicating that an increased seizure burden may lead to poorer responses to standard treatments, necessitating more aggressive or alternative therapeutic approaches [2,3].

These outcomes underscore the importance of individualised treatment plans based on the specific clinical scenario of each neonate, considering seizure frequency and the risk of adverse effects. The apparent parity in efficacy between LEV and PB does not diminish the clinical utility of either drug; rather, it highlights the need for a more stratified approach to ASM selection tailored to the nuanced needs of neonatal patients.

In analysing the determinants of antiseizure medication (ASM) efficacy, our study found a notable variation in response correlating with seizure frequency among neonatal subjects. Term neonates, constituting the majority of the first-line treatment group, demonstrated an ASM response that intensified with seizure frequency. Specifically, rare seizures resulted in an incidence rate ratio (IRR) of 2.09, whereas more frequent seizures and status epilepticus exhibited higher IRRs of 3.25 and 4.01, respectively. This suggests that an increased seizure load may positively influence the therapeutic response to initial ASM intervention. This aligns with the established understanding that the higher the frequency of seizures, the more challenging it is to achieve control. This is supported by various studies, including those by Sharpe et al. [5] and Glass et al. [12], which highlighted the complexity of treating frequent status epilepticus seizures in neonates.

For the second-line treatment cohort, our analysis revealed a slight but noteworthy improvement in treatment outcomes when switching to phenobarbital (PB), as evidenced by an odds ratio marginally above one (approximately 1.09). This indicates that PB may offer incremental benefits over LEV in instances where the initial treatment with LEV does not achieve the desired seizure control. These findings suggest that seizure frequency is a key factor in the selection and evaluation of the efficacy of ASM. This is consistent with meta-analyses that prove that second-line ASMs are crucial when first-line treatment is not fully effective [3,11,17].

The absence of a significant difference in the response to ASMs according to gestational age and aetiology points to potential uniformity in the approach to managing various seizure aetiologies in neonates. However, it is critical to note that while statistical models can show associations, they do not necessarily prove causation or the best treatment path for all patients, as individual responses can vary widely.

This study suggests that while PB could be the traditional mainstay of treatment, LEV is emerging as a potential first-line treatment owing to its favourable safety profile. Again, PB is highly preferred as a second-line treatment because of its high effectiveness in seizure sessions.

This is supported by a systematic review by McHugh et al. [13] and a meta-analysis by Qiao et al. [2], which compared the efficacy of LEV and PB in neonatal seizures. Thus, the clinical decision-making process should be tailored considering the individual patient’s seizure frequency, potential side effects, and total response to the initial treatment.

## 5. Conclusions

This study systematically evaluated the efficacy and safety of PB and LEV as the first- and second-line treatment for seizures in a cohort of 104 neonates. Although LEV is commonly used as the initial treatment, it did not completely control seizures in nearly half of the treated neonates. The secondary application of LEV and PB demonstrated mixed results, with some patients requiring further medication, suggesting the complexity of achieving seizure control in neonates.

The study also underscored the safety profiles of ASMs, with LEV showing a significantly lower incidence of adverse events than PB.

These findings prompt a critical review of the treatment protocols for neonatal seizures, emphasising the importance of individualised approaches. Although the mortality rates differed between the treatment groups, this difference further highlights the need for careful consideration of each neonate’s specific circumstances when deciding on a treatment plan.

This analysis suggests that while PB remains a traditional option in neonatal seizure management, LEV has emerged as a potentially safer and equally effective first-line treatment. This study adds to the existing body of evidence, necessitating a balance between efficacy and safety in choosing the appropriate ASM and calls for ongoing research to refine therapeutic strategies for this vulnerable population.

## Figures and Tables

**Table 1:** Assessment of neonatal seizure management: comparative clinical outcomes between levetiracetam and phenobarbital treatment.

Clinical Features	PB as First-Line ASM, n = 71 (68.26%) *	LEVas First-Line ASM, n = 33 (31.74%) *	*p*-Value	All Neonates, n = 104 (100%) *
Sex			0.434	
Male	19 (26.76%)	40 (38.46%)		59 (56.73%)
Female	15 (21.13%)	30 (28.85%)		45 (43.27%)
Birth weight (g)	1791.02 (±1072.49)	1527.79 (±1069.48)	0.264	1712.65 (±1077.59)
Gestational week	31.61 (±5.80)	30.12 (±6.30)	0.258	31.05 (±5.96)
Hospitalisation (days)	62.37 (±51.22)	73.82 (±50.28)	0.306	67.09 (±50.75)
Maternal age (years)	32 (19–42)	32 (19–40)	0.075	31.5 (18–42)
Mode of delivery			0.721	
NSD	24 (33.80%)	15 (45.45%)		39 (37.50%)
C/S	40 (56.34%)	24 (72.73%)		64 (61.54%)
Prenatal complication				
Early membrane rupture	7 (9.86%)	4 (12.12%)	0.783	11 (10.58%)
IUGR	7 (9.86%)	2 (6.06%)		9 (8.65%)
preeclampsia	8 (11.27%)	1 (3.03%)		9 (8.65%)
chorioamnionitis	5 (7.04%)	1 (3.03%)		6 (5.77%)
Etiology of the Seizure			0.682	
Hypoxic-ischemic	24 (33.80%)	10 (30.30%)		34 (32.69%)
Structural: vascular	18 (25.35%)	8 (24.24%)		26 (25.00%)
Structural: brain malformation (Genetic)	8 (11.27%)	3 (9.09%)		11 (10.58%)
Infectious	6 (8.45%)	5 (15.15%)		11 (10.58%)
Metabolic	0 (0%)	1 (3.03%)		1 (0.96%)
Unknown	13 (18.31%)	6 (18.18%)		19 (18.27%)
Day of seizure onset	13.09 (±22.29)	11.52 (±21.77)	0.74	12.31 (±22.03)
Seizure frequency			0.042	
Rare	13 (18.31%)	23 (69.70%)		36 (34.61%)
Frequent	14 (19.72%)	32 (97.00%) *		46 (44.23%)
Status epilepticus	5 (7.04%)	17 (51.52%)		22 (21.15%)
Response to treatment within first line therapy			0.051	
Incomplete	15 (21.13%)	47 (47.47%)		62 (59.61%)
Complete	17 (23.94%)	25 (75.76%)		42 (40.38%)
Outcome			0.309	
Discharged	48 (67.61%)	25 (75.76%)		73 (70.19%)
Died	16 (22.54%)	15 (45.45%)		31 (29.81%)

ASM, anti-seizure medication; C/S, caesarean section; IUGR, intrauterine growth retardation; LEV, levetiracetam; N, number of patients; NSD, normal spontaneous delivery; PB, phenobarbital. Statistics of the cohort, including counts and percentages for categorical variables and mean and range for continuous variables. Percentages are given in the columns. * n (%); mean (range); median (±SD); Wilcoxon rank sum test; Fisher’s exact test.

**Table 2:** Multivariate analysis of determinants of ASM response in neonatal seizure management.

	First-Line ASM (n = 104)				Second-Line ASM (n = 62)			
Clinical Features	N	IRR	95% CI	*p*-Value	N	IRR	95% CI	*p*-Value
Gestational Age								
Term	79	_	_		45	_	_	
Preterm	25	0.90	0.59, 1.57	0.79	17	0.99	0.50, 1.79	0.86
Etiology								
Hypoxic-ischemic	34	_	_		25	_	_	0.78
Structural: vascular	26	1.04	0.70, 1.59	0.67	18	0.79	0.44, 1.48	0.58
Structural: brain malformation and Genetic	12	0.09	0.39, 2.27	0.28	10	0.49	0.17, 1.56	0.71
Infectious	11	1.29	0.81, 1.88	0.48	8	1.04	0.17, 1.56	0.98
Metabolic	1	1.35	0.51, 3.26	0.53	1	0.91	0.16, 2.92	
Seizure Frequency								
Rare seizures	36	2.09	1.19, 3.98	0.010	13	3.01	0.75, 18.9	0.18
Frequent seizures	46	3.25	1.87, 5.69	<0.001	32	4.25	1.39, 26.9	0.01 *
Status epilepticus	22	4.01	2.08, 5.97	<0.001	17	6.78	1.91, 34.2	0.02 *
First-Line ASM								
PB	71	_	_	_				
LEV	33	0.96	0.69, 1.53	0.98				
Second Line ASM								
PB					20	1.09	0.69, 2.09	0.04
LEV					42			

ASM, antiseizure medication; LEV: levetiracetam; N, number of patients; PB: phenobarbital. In a fitted regression model including gestational age, aetiology, seizure frequency, and ASM, only seizure frequency remained significantly associated with response to first- and second-line ASMs (*p* < 0.05). In the first-line ASM column, 13 patients with unknown seizure aetiology were excluded. In the second-line ASM column, only patients who received PB or LEV as second-line ASM were included. * Statistically significant at *p* < 0.05.

## Data Availability

The data presented in this study are available via communicaiıon with the corresponding author.
